# Poly (adenosine diphosphate [ADP]–ribose) polymerase (PARP) inhibitors as maintenance therapy in women with newly diagnosed ovarian cancer: a systematic review and meta-analysis

**DOI:** 10.1007/s00404-021-06070-2

**Published:** 2021-05-21

**Authors:** Hongyan Cheng, Junjun Yang, Huixin Liu, Yang Xiang

**Affiliations:** 1 Department of Obstetrics and Gynecology, Peking Union Medical College Hospital, Chinese Academy of Medical Sciences and Peking Union Medical College, No.1 Shuaifuyuan, Wangfujing, Dongcheng District, Beijing, 100730 People’s Republic of China; 2 Department of Clinical Epidemiology and Biostatistics, Peking University People’s Hospital, No.11 Xizhimen South Street Xicheng District, Beijing, 100044 People’s Republic of China

**Keywords:** Poly (adenosine diphosphate [ADP]–ribose) polymerase (PARP) inhibitors, Newly diagnosed ovarian cancer, Systematic review, Meta-analysis

## Abstract

**Purpose:**

To investigate the efficacy and safety of poly (adenosine diphosphate [ADP]–ribose) polymerase (PARP) inhibitors (including their different types) as maintenance therapy in women with newly diagnosed ovarian cancer, and to explore whether this therapy produces a survival benefit in a subgroup population with specific clinical characteristics.

**Methods:**

We searched MEDLINE, EMBASE, the Cochrane Library, Web of Science and relevant clinical research registry platforms on October 1, 2019, and included randomized controlled trials (RCTs) that compared PARP inhibitors with placebo in women (aged ≥ 18 years) with newly diagnosed epithelial ovarian cancer.

**Results:**

We identified four RCTs with 3,070 participants. Compared with placebo, PARP inhibitor maintenance therapy showed a clinically significant benefit on progression free survival (PFS) in homologous recombination deficiency (HRD) positive population (hazard ratio [HR], 0.39; 95% confidence interval [CI], 0.29–0.53). In contrast, no clear differences were identified between the groups in the HRD negative population (HR, 0.83; 95% CI 0.67–1.03). Further, there was no clear difference between the groups in terms of other outcomes (overall survival, health-related quality of life, and adverse events).

**Conclusions:**

PARP inhibitor maintenance therapy significantly prolongs the PFS of patients with newly diagnosed ovarian cancer, especially in HRD positive patients. The diagnostic test used to determine HRD status plays an important role in guiding PARP inhibitor maintenance therapy. Compared with placebo, the effect of PARP inhibitors on ovarian cancer was probably not affected by the International Federation of Gynecology and Obstetrics stage status, response to first-line chemotherapy, and residual macroscopic disease after debulking surgery.

**Supplementary Information:**

The online version contains supplementary material available at 10.1007/s00404-021-06070-2.

## Introduction

Ovarian cancer is the second most common cause of death from gynecologic malignancies worldwide. It is estimated to be diagnosed in more than 239,000 women per year globally with approximately 152,000 deaths annually [1, 2]. Women with ovarian cancer have not experienced the same 20% increase in 5-year survival that advances in screening and therapies have brought to many other patients with cancer [3]. At present, ovarian cancer has an average 5-year survival rate of approximately 47% after diagnosis [2]. Although early-stage disease is highly curable [4], the majority of women present with advanced stage (III/IV) disease and more than 75% of women with late-stage ovarian cancer succumb to the disease [5–7].

Surgical cytoreduction and platinum-taxane combination chemotherapy have been the mainstay therapies for decades in patients with ovarian cancer. Further, antiangiogenic agents, such as bevacizumab, have been widely used in patients with suboptimal debulking or stage IV disease [8]. However, despite optimal surgery and advances in chemotherapy regimens, 75% of women with ovarian cancer experience relapse within 3 years after diagnosis [9].

The introduction of poly (adenosine diphosphate [ADP]–ribose) polymerase (PARP) inhibitors has led to major improvements in the use of maintenance therapy for women with advanced high-grade serous ovarian cancer [10]. PARP enzymes (PARP1 and PARP2) play a vital role in the repair of deoxyribonucleic acid (DNA) single-strand breaks (SSBs) through several repair pathways, one of which is homologous recombination [11]. PARP inhibitors block SSB repair by trapping the enzymes onto the DNA leading to the accumulation of double-stranded DNA breaks which, when not repaired, result in cell death [12]. Ovarian cancer cells with homologous recombination deficiencies (HRDs), including breast cancer gene (BRCA) 1/2 mutations, are particularly sensitive to the effects of PARP inhibitors owing to the enhanced cytotoxicity arising from the harmful effect of an additional non-functioning gene, a phenomenon referred to as “synthetic lethality” [13, 14].

Several studies have demonstrated the efficacy of PARP inhibitors in women with and without BRCA1/2 germline mutations, particularly when they are used as both single agents and maintenance therapy for the treatment of platinum-sensitive recurrent serous ovarian cancer with consistent findings of significant increases in progression-free survival (PFS) [12, 15–20]. Based on these findings, the benefits of PARP inhibitors have been well demonstrated in women with recurrent disease, and PARP inhibitors (e.g., olaparib, rucaparib, and niraparib) have been approved for the treatment of high-grade epithelial ovarian cancer. Furthermore, four studies [21–24] showed the benefits of PARP inhibitors as first-line maintenance therapy. Based on these findings, niraparib has been approved as first-line monotherapy maintenance in patients with advanced ovarian cancer regardless of biomarkers status [25, 26]. Moreover, Olaparib has been approved as maintenance therapy in patients with deleterious BRCA mutations after a response to first-line chemotherapy, and olaparib plus bevacizumab has been approved as maintenance therapy in patients with HRD positive ovarian cancer [23]. There are some differences in these studies [21–24], such as participants, subgroup factors, HRD cut-off, different control group and treatment regimens. The benefits of PARP inhibitors remain unclear, which may lead to different clinical decisions in practice.

In light of these findings, the primary objective of this systematic review was to determine the benefits and harms of PARP inhibitors (including their different types) as maintenance therapy in patients with newly diagnosed ovarian cancer. The secondary objective was to explore whether PARP inhibitor maintenance therapy produces a survival benefit in the following subgroup population:

- patients with the International Federation of Gynecology and Obstetrics (FIGO) stage III or IV;
- patients with complete response (CR) or partial response (PR) after first-line chemotherapy;
- patients presenting with residual macroscopic disease after debulking surgery.

## Materials and methods

We registered the review protocol with PROSPERO (CRD42020154131). This systematic review and meta-analysis was performed in compliance with the methodological standards described in the Cochrane Handbook of Interventional Reviews and was reported in accordance with Preferred Reporting Items for Systematic Reviews and Meta-Analyses (PRISMA) standards [27].

### Search strategy

We searched electronic databases (including MEDLINE, EMBASE, the Cochrane Library, and Web of Science) on October 1, 2019, using the keywords ovarian neoplasms, olaparib, niraparib, veliparib, and placebo. Further, we searched the metaRegister of Controlled Trials (www.controlled-trials.com/mrct/), ClinicalTrials.gov (ClinicalTrials.gov), and World Health Organization International Clinical Trials Registry Platform (apps.who.int/trialsearch/) to identify additional published or unpublished data. There were no limitations on language, date, document type, or publication status. The detailed search strategies are presented in Online Resource Methods. We manually searched the references of relevant systematic reviews to identify additional randomized controlled trials (RCTs) for inclusion.

### Eligibility criteria

RCTs fulfilling the following criteria were included: (1) participants were female (aged ≥ 18 years) with newly diagnosed epithelial ovarian cancer, diagnosed via any appropriate diagnostic criteria, regardless of disease stage, BRCA-mutation or HRD status; and (2) the comparison was PARP inhibitors versus placebo, of which, PARP inhibitors were used as first-line maintenance therapy (for participants who had CR or PR after chemotherapy). The primary outcome was PFS as assessed by the investigators. Where those data were unavailable, we extracted data assessed by blinded independent central review instead. Secondary outcomes were overall survival (OS), health-related quality of life (HRQoL), and adverse events (AEs, overall or specific).

### Study selection

**Fig. 1:**
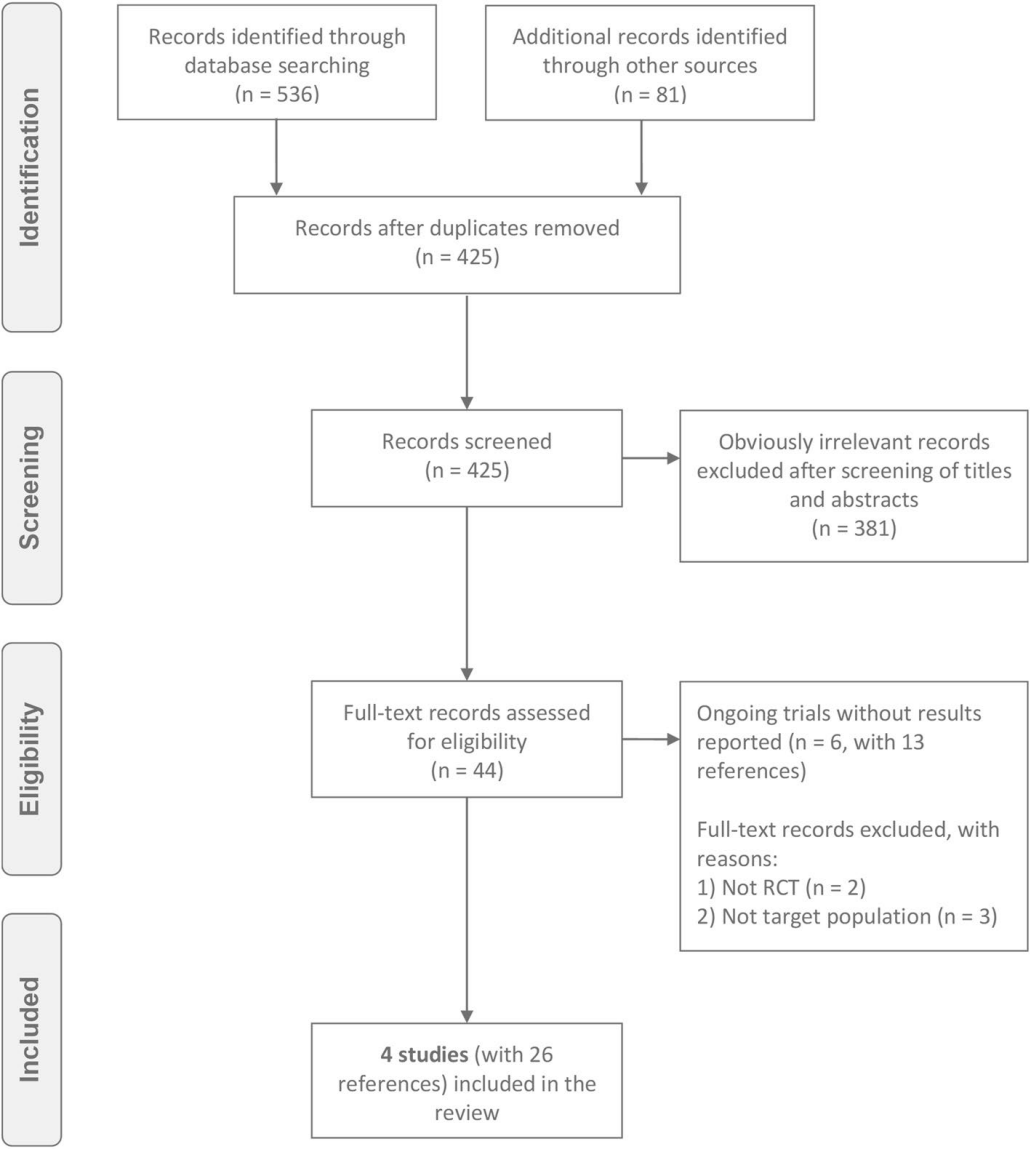
Study flow diagram

Two reviewers independently investigated the search results. After removing duplicate records and initial screening of all remaining references via titles and abstracts, the full text reports of the references appear to fulfill the inclusion criteria were obtained and further scrutinized to finalize the inclusion decision. Any disagreement was resolved through discussion by two reviewers with assistance from a third reviewer if necessary. Figure 1 presents the PRISMA flow diagram to illustrate the study selection process.

### Data extraction

Two reviewers independently extracted the qualitative and quantitative data using a standardized data collection form (Online Resource Table 1). Any disagreements were resolved via discussions with assistance from a third reviewer if necessary.

### Quality assessment

We evaluated every domain of risk of bias using the Cochrane risk of bias tool for RCTs, including sequence generation, allocation concealment, blinding of participants and personnel, blinding of outcome assessment, incomplete outcome data, selective outcome reporting, and other bias [28]. We generated a ‘risk of bias graph’ and a ‘risk of bias summary’. Then, we assessed the quality of evidence using the GRADE approach [29].

### Statistical analysis

We summarized outcomes using hazard ratios (HRs) for time-to-event data, risk ratios (RRs) for other dichotomous data, and mean differences (MDs) or standardized mean differences (SMDs) with 95% confidence intervals (CIs) for all continuous outcome data. We used a random effect model for all meta-analyses using RevMan 5.3 [30]. If a meta-analysis was considered inappropriate, we described the outcome data narratively. To explore clinical heterogeneity, we performed priori subgroup analyses of the primary outcome based on the following characteristics: (1) BRCA-mutation status (positive versus negative); (2) HRD status (positive versus negative); (3) FIGO stage (III versus IV); (4) response to first-line chemotherapy (CR versus PR); (5) with or without residual macroscopic disease after debulking surgery We performed sensitivity analyses focus on PARP inhibitors monotherapy versus placebo to test the robust of results of the primary outcome. Statistical heterogeneity was estimated via *I*^2^ and *χ*^2^ statistics (substantial statistical heterogeneity is defined as *I*^2^ ≥ 50% with a *P* value of *χ*^2^ test < 0.1).

## Results

### Study selection

The initial search retrieved a total of 536 references through databases and 81 references through manual search of the reference lists of relevant reviews and included studies. After deduplication, 425 unique references remained. We excluded 381 references after inspecting their titles and abstracts. We completely read the remaining 44 references and subsequently excluded 18 references due to the reasons (provided in Fig. 1). Finally, we included four studies [21–24] (with 26 references) in the review.

### Study characteristics

A total of four RCTs (PAOLA-1 [24], PRIMA [22], SOLO1 [23], and VELIA [21]) with 3,070 participants were included. The sample sizes ranged from 391 [23] to 806 [24]. All RCTs were multicenter studies, with participants from Australia, Austria, Belgium, Brazil, Canada, China, Czech Republic, Denmark, Finland, France, Germany, Hungary, Ireland, Israel, Italy, Japan, Korea, Netherlands, New Zealand, Norway, Poland, Romania, Russia, Serbia, Spain, Sweden, Switzerland, Ukraine, the United Kingdom, and the United States.

The participants aged 22–88 years, with a median age of 61 or 62 years (SOLO1 [23] reported the mean age as 53.5 years). The participants were all newly diagnosed with ovarian cancer, advanced FIGO stage III (*n* = 2240) to IV (*n* = 828) (two missing data), and high-grade epithelial serous or endometrioid ovarian, fallopian tube, or peritoneal cancer. 87.1% of participants had an Eastern Cooperative Oncology Group (ECOG) score of 0–1 (2625/3015). As reported, 37.4% of participants had a BRCA mutation, and 45.2% of participants were HRD positive (Table 1).

**Table 1:** Included RCTs (multi-center) comparing PARPi to placebo in patients with newly diagnosed ovarian cancer

Author with publication year (Trial ID)	Sample size, *n* (PARPi/Placebo)	Stage of disease [No. (%)]	BRCA-mutation status [No. (%)]	HRD status [No. (%)]	Target outcomes^a^
Ray-Coquard 2019^b^ (PAOLA-1, NCT02477644)	806 (Olaparib 537/269)	FIGO stage III 564 (70) IV 242 (30)	Positive 241 (29.9) Negative or Unknown 565 (70.1)	Positive 387 (48.0) Negative 277 (34.4) Unknown 142 (17.6)	PFS; HRQoL; AE
Moore 2018 (SOLO1, NCT01844986)	391 (Olaparib 260/131)	FIGO stage III 325 (83.1) IV 66 (16.9)	Positive 391 (100)	Not reported	PFS; OS; HRQoL; AE
González-Martín 2019 (PRIMA, NCT02655016)	733 (Niraparib 487/246)	FIGO stage III 476 (64.9) IV 257 (35.1)	of patients with HRD (*n* = 373): Positive 223 (30.4) Negative 150 (20.5) Unknown 360 (49.1)	Positive 373 (50.9) Negative or Unknown 360 (49.1)	PFS; HRQoL; OS; AE
Coleman 2019^c^ (VELIA, NCT02470585)	1140 (Veliparib 382/758)	FIGO stage III 875 (76.8) IV 263 (23.1) Missing data 2 (0.1)	Positive 298 (26.1) Negative 742 (65.1)	Positive 627 (55) Negative 372 (32.6) Unknown 141 (12.4)	PFS; HRQoL; AE

*CA-125* cancer antigen 125, *CR* complete response, *PR* partial response, *PFS* progression free survival, *OS* overall survival, *HRQoL* health-related quality of life, *AE* adverse event, *FIGO* International Federation of Gynecology and Obstetrics

^a^OS unable to use in PAOLA-1 and VELIA trials due to not sufficiently mature

^b^Bevacizumab combined use in both olaparib and placebo groups

^c^Three compared groups in this RCT: (1) control group: chemotherapy plus placebo followed by placebo maintenance; (2) veliparib-combination-only group: chemotherapy plus veliparib followed by placebo maintenance; (3) veliparib-throughout group: chemotherapy plus veliparib followed by veliparib maintenance. Although the results contained a combination of ‘treatment phase’ and ‘maintenance treatment phase’ (no separate data available for ‘maintenance treatment phase’, i.e. group 2 versus group 1), data on group 3 versus group 1 were used in meta-analysis based on conclusions of this trial which PFS in group 2 does not differ from that in group 1

All the participants in the four RCTs had received platinum-based chemotherapy, and 87.8% of the participants (2698/3070) had undergone debulking surgery (primary: 66.4%, 2040/3070; interval: 21.4%, 658/3070). The inclusion criteria of these studies were that participants must have achieved CR or PR with no clinical evidence of disease progression on the posttreatment scan or indications of an increase in CA-125 level following completion of this chemotherapy course (the details of CA-125 assessment can be found in Online Resource Table 2). SOLO1 [23], which only included participants with BRCA 1/2 germline mutations, compared olaparib (300 mg twice daily) with placebo. PRIMA [22], which only included participants with high risk, compared niraparib (300 mg once daily) with placebo. VELIA [21] compared veliparib plus chemotherapy (400 mg twice daily) with placebo plus chemotherapy (contained a combination of ‘treatment phase’ and ‘maintenance treatment phase’, details were shown in Table 1). And PAOLA-1 [24] compared olaparib (300 mg twice daily) plus bevacizumab (15 mg/kg, every 3 weeks) treatment with bevacizumab treatment alone.

### Risk of bias in included studies

The overall risk of bias in all included RCTs was low (Online Resource Fig. 1). All the RCTs adequately reported random sequence generation and allocation concealment. Blinding of the participants and personnel was ensured in all studies. Missing data were well-addressed in all RCTs, and all the outcomes predefined in the protocol were well-reported. Further, all RCTs were funded by the industry. We rated the other bias of these RCTs as unclear risk, except for the PAOLA-1 trial [24], which was an academic-sponsored study.

### PFS

Four RCTs (PAOLA-1 [24], PRIMA [22], SOLO1 [23], and VELIA [21]) reported PFS as assessed by investigators and were included in meta-analysis. We did not pool the data for the overall population due to excessive heterogeneity (Online Resource Fig. 2), but reported the estimated effect in the planned subgroup population.

**Fig. 2:**
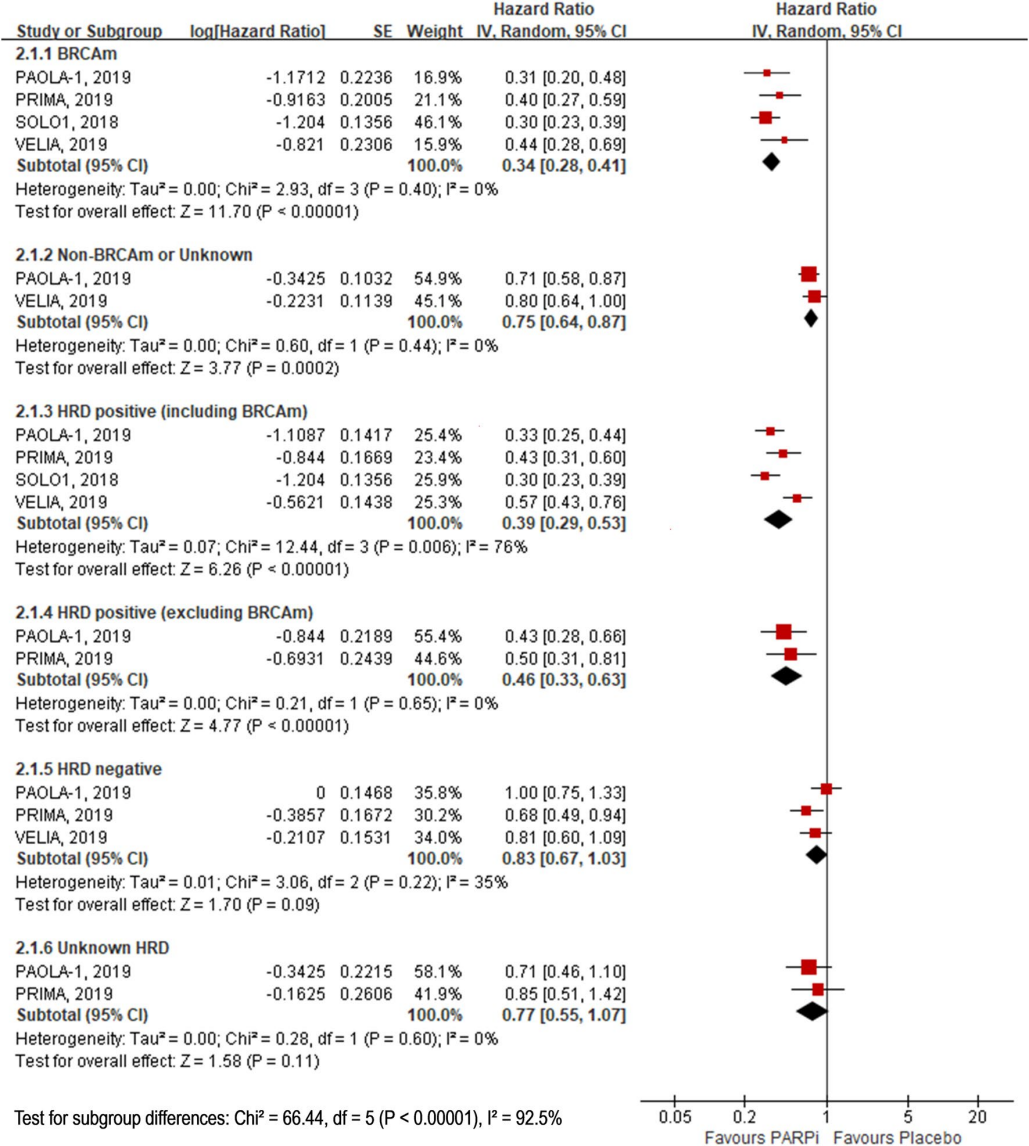
Subgroup analysis for comparison of PFS between PARPi and placebo. *PFS* progression free survival, *PARPi* poly (adenosine diphosphate [ADP]–ribose) polymerase inhibitors, *BRCAm* Breast cancer gene mutation, *HRD* Homologous recombination deficiencie

The results showed that participants who received PARP inhibitors had longer PFS than those who received placebo in the BRCA mutation population (HR, 0.34; 95% CI 0.28–0.41), non-BRCA mutation population (HR, 0.75; 95% CI 0.64–0.87), HRD positive (including BRCA mutation) population (HR, 0.39; 95% CI 0.29–0.53), and HRD positive (excluding BRCA mutation) population (HR, 0.46; 95% CI 0.33–0.63). However, in the HRD negative population, there were no clear difference between the groups (HR, 0.83; 95% CI 0.67–1.03). (Fig. 2 and Online Resource Table 3).

**Fig. 3:**
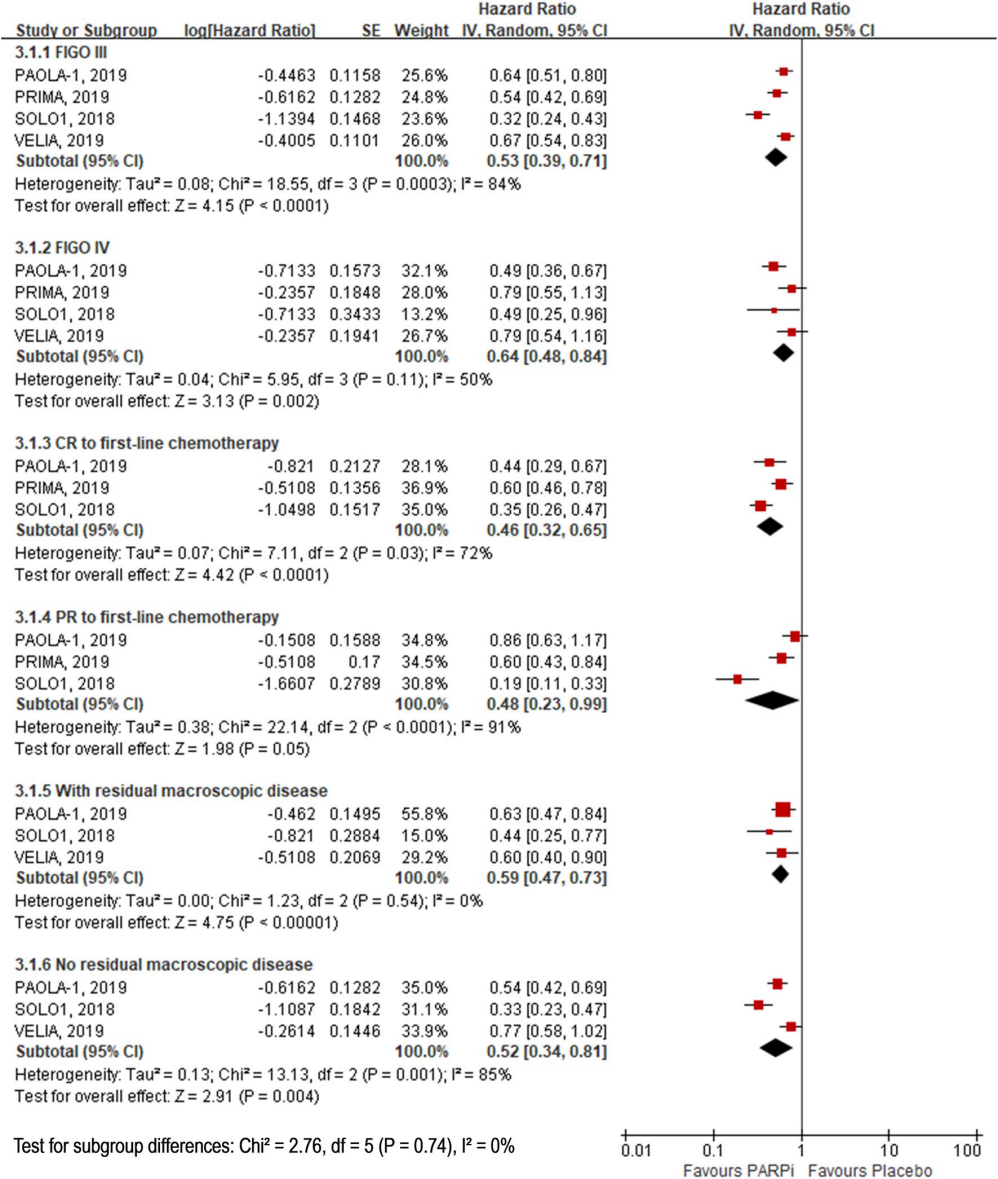
Subgroup analysis for comparison of PFS between PARPi and placebo. *PFS* progression free survival, *PARPi* poly (adenosine diphosphate [ADP]–ribose) polymerase inhibitors, *FIGO* International Federation of Gynecology and Obstetrics, *CR* complete response, *PR* partial response, *HR* hazard ratio. Note: Bevacizumab combined use in both olaparib and placebo groups in PAOLA-1 trial. Chemotherapy combined use in both veliparib and placebo groups in VELIA trial

The participants who received PARP inhibitors had longer PFS than those who received placebo irrespective of FIGO disease stage (III: HR, 0.53; 95% CI 0.39–0.71; and IV: HR, 0.64; 95% CI 0.48–0.84). PFS was longer in the PARP inhibitor group than in the placebo group for participants with CR to first-line chemotherapy (HR, 0.46; 95% CI 0.32–0.65) as well as for those with PR to first-line chemotherapy (HR, 0.48; 95% CI 0.23–0.99). Similar results were obtained in the presence of residual macroscopic disease after debulking surgery performed before trial enrollment (with residual macroscopic disease: HR, 0.59; 95% CI 0.47–0.73; and no residual macroscopic disease: HR, 0.52; 95% CI 0.34–0.81). (Fig. 3 and Online Resource Table 3).

Sensitivity analyses which only focus on PARP inhibitors monotherapy (PRIMA [22], SOLO1 [23]) was performed and achieved similar results, although the PRIMA trial [22] showed that participants who received PARP inhibitors had longer PFS than those who received placebo in HRD negative population (HR, 0.68; 95% CI 0.49–0.94), and no clear difference was found in participants with FIGO stage IV (HR, 0.79; 95% CI 0.55–1.13). (Online Resource Fig. 3 and Online Resource Fig. 4).

### OS

Two RCTs (PRIMA [22], and SOLO1 [23]) reported OS (*n* = 1124) and were included in the meta-analysis. However, the data on OS in these two RCTs were insufficient (the median OS was not reached; death in PRIMA [22]: 10.8%; death in SOLO1 [23]: 21%). Based on interim analysis of these two RCTs, the results of the meta-analysis demonstrated no clear differences in OS between the groups (HR, 0.82; 95% CI 0.59–1.13, Online Resource Fig. 5) with low-quality of evidence (Online Resource Table 3).

The other two RCTs (PAOLA-1 [24], and VELIA [20, 21]) also reported that they did not perform an analysis due to insufficient data (the duration of follow-up was too short to assess this outcome).

### HRQoL

Four RCTs (PAOLA-1 [24], PRIMA [22], SOLO1 [23], and VELIA [21]) reported HRQoL using different scales, covering key physical and functional well-being, and symptoms related to disease or treatment (Online Resource Text). In PAOLA-1 [24] (*n* = 744), HRQoL was assessed as the adjusted mean change from baseline in the global health status-QoL score of the European Organization for Research and Treatment of Cancer (EORTC) Quality of Life Questionnaire (EORTC QLQ-C30; the scores ranged from 0 to 100, with a higher score indicating a better outcome, and a minimal clinically important difference defined as 10 points). In SOLO1 [23] (*n* = 362), HRQoL was assessed as the change from baseline in the Trial Outcome Index (TOI) score of the Functional Assessment of Cancer Therapy-Ovarian Cancer (FACT-O) questionnaire (scores ranged from 0 to 100, with a higher scores indicating a better outcome and a difference of 10 points indicating a clinically meaningful difference). High-quality evidence (Online Resource Table 2) indicated no clear difference in HRQoL scores between the groups (SMD, − 0.12; 95% CI − 0.60 to 0.36, Online Resource Fig. 6). The results also showed significant heterogeneity probably because SOLO1 [23] included all participants with BRCA mutation receiving olaparib only, whereas PAOLA-1 [24] included the overall population receiving olaparib combined with bevacizumab. However, the change score reflecting HRQoL in both trials indicated that neither the PARP inhibitor nor placebo groups achieved meaningful clinical changes, and that there was no clinically meaningful difference between the groups in terms of patients’ HRQoL (Online Resource Fig. 6). The quality of evidence was high (Online Resource Table 3).

The PRIMA trial [22] reported the change in score from baseline of the Functional Assessment of Cancer Therapy-Ovarian Symptoms Index (FOSI) (detailed data not reported in this trial), which also showed no clear differences in participants’ HRQoL between the two compared groups.

The VELIA trial [21] reported HRQoL, which was assessed as the mean change from baseline in a subset of National Comprehensive Cancer Network Functional Assessment of Cancer Therapy Ovarian Symptom Index–18 (NFOSI-18)—Disease Related Symptom scores [31]. The differences in the mean change from baseline between the groups were trivial (range 0.0–2.1) and were not considered to be clinically meaningful.

### AEs

**Fig. 4:**
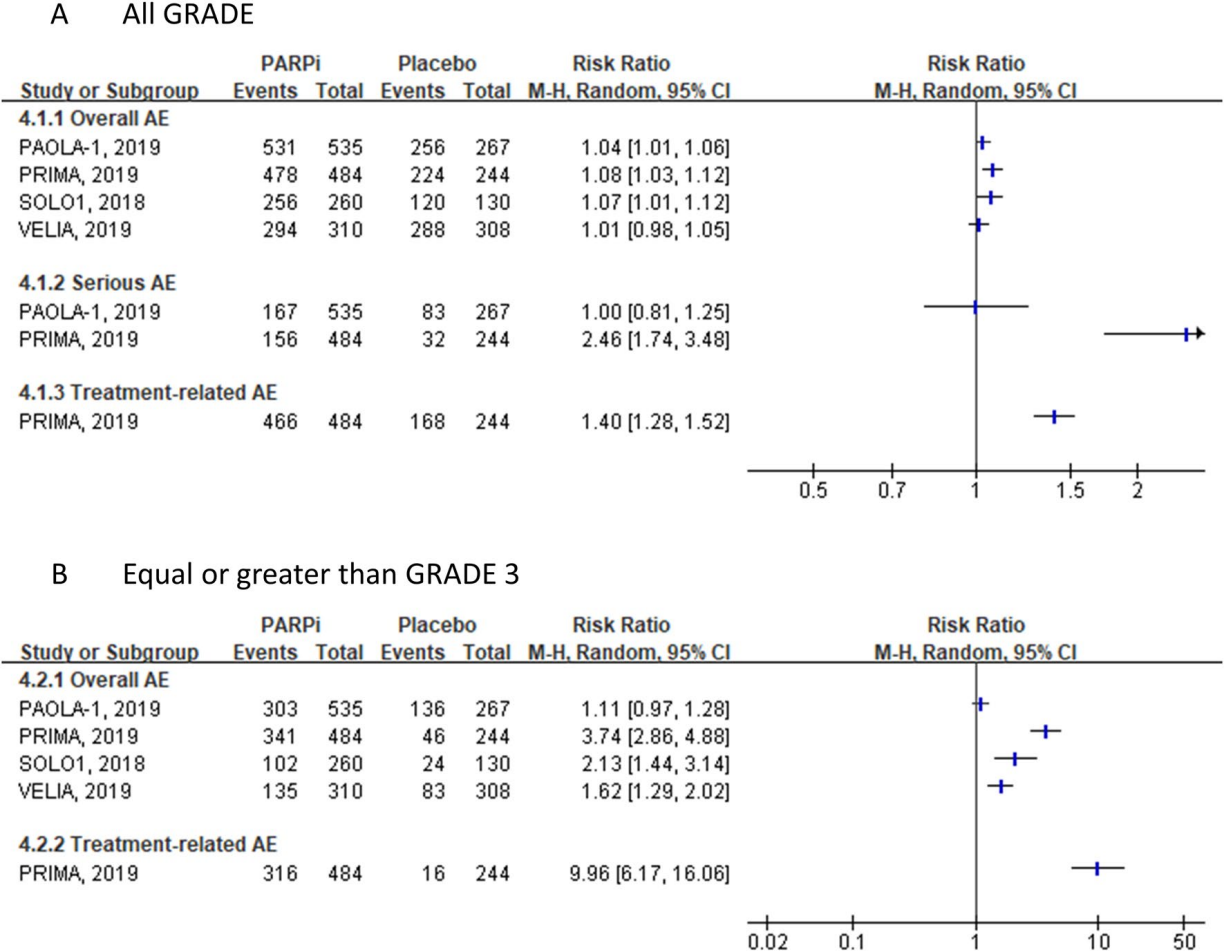
Comparison of AEs between PARPi and placebo in each included study. *AEs* adverse events, *PARPi* poly (adenosine diphosphate [ADP]–ribose) polymerase inhibitors; *RR* risk ratio. Note: Bevacizumab combined use in both olaparib and placebo groups in PAOLA-1 trial. Chemotherapy combined use in both veliparib and placebo groups in VELIA trial

All the four included RCTs (PAOLA-1 [24], PRIMA [22], SOLO1 [23], and VELIA [21]) (*n* = 2538) reported AEs. Considering that AEs vary across various types of PARP inhibitors, we did not perform meta-analysis with this outcome. The results of all the included RCTs showed that PARP inhibitors did not have a clinically important impact on the overall incidence of AEs at 2- or 3-year follow-up. Only one RCT (PRIMA [22]) reported the total number of treatment-related AEs, and found that more participants experienced treatment-related AEs (any grade or grade ≥ 3) in the niraparib group than in the placebo group. In contrast, PAOLA-1 [24] found that compared to using bevacizumab alone, olaparib combined with bevacizumab did not increase the risk of serious AEs (any grade or grade ≥ 3). (Fig. 4).

The specific AEs that occurred during PARP inhibitor maintenance therapy in each RCT are shown in Online Resource data (Online Resource Table 4, and Online Resource Table 5). In all the four RCTs, the most common AE in the PARP inhibitor group was nausea (over 50%) with most events (approximately 95%) less than grade 3. Low-grade abdominal pain, arthralgia, constipation, diarrhea and headache were also common in the compared groups. The most common grade 3 or higher AEs in the PARP inhibitor group were primarily related to the hematological system, namely thrombocytopenia, anemia and neutropenia. The results also showed that many more events occurred in the niraparib group in the PRIMA [22] trial (*n* = 484) than in other RCTs using other types of PARP inhibitors (grade ≥ 3: thrombocytopenia, 41.7%; anemia, 40.0%; and neutropenia, 20.5%; Online Resource Table 5). However, in the PAOLA-1 [24] trial, fewer participants in the olaparib combined with bevacizumab group experienced hypertension with grade ≥ 3 (18.7%, 100/535) than those in the placebo combined with bevacizumab group (30.3%, 81/267). A summary of the other specific AEs reported by a single trial is presented in Table 2.

**Table 2:** PARPi versus placebo: the specific adverse events which only reported in the specific trial

Trail ID	Specific adverse events
PAOLA-1 (Olaparib + bevacizumab)	lymphopenia^a^, urinary tract infection^a^, musculoskeletal pain^a^, edema^a^, stomatitis or pharyngitis^a^, renal impairment, proteinuria^a^, intestinal obstruction^a^, cystitis, sleep disorder^a^, muscle spasms, rash^a^, erythema, neck pain, coronary artery disease^a^, pyelonephritis, cardiac failure^a^, acute lymphocytic leukaemia, acute pyelonephritis, erythematous rash, kidney infection, lung cancer, myeloma, pancreatic cancer, pruritic rash, squamous skin cancer, umbilical erythema, balance disorder, pyuria, urosepsis, dysgeusia^b^
SOLO1 (Olaparib)	upper abdominal pain, dyspepsia, head and neck cancer
PRIMA (Niraparib)	platelet count decreased^a^, neutrophil count decreased^a^, asthenia^a^, white blood cell count decreased^a^, hot flush^a^, abdominal distension, blood creatinine increased^b^, cough^b^
VELIA (Veliparib + chemotherapy)	back pain^b^

^a^All GRADE including GRADE >  = 3

^b^GRADE >  = 3

Regarding other AEs of interest, myelodysplastic syndrome, acute myelocytic leukemia or aplastic anemia occurred in 11 participants in the PARP inhibitor group (6 in the PAOLA-1 [24] trial, 3 in the SOLO1 [23] trial, 1 in each of the other two trials) and in 2 participants in the placebo group (1 in the PAOLA-1 [24] trial and 1 in the SOLO1 [23] trial). New primary cancers (including acute lymphocytic leukemia, head and neck cancer, lung cancer, myeloma, pancreatic cancer, squamous cell skin cancer, breast cancer, and thyroid cancer) mainly occurred in the olaparib group (7 in the PAOLA-1 [24] trial and 5 in the SOLO1 [23] trial). Pneumonitis or interstitial lung disease manifested in six participants in the olaparib combined with bevacizumab group (PAOLA-1 [24]), five participants in the olaparib alone group (SOLO1 [23]), and no participant in the bevacizumab or placebo group. (detailed data are shown in Online Resource Table 4 and Online Resource Table 5).

## Comment

This systematic review summarized four RCTs with 3070 participants, with the aim of assessing the efficacy and safety of PARP inhibitors as maintenance therapy in patients with newly diagnosed ovarian cancer. The results of the meta-analysis indicated that compared with placebo, PARP inhibitor maintenance therapy is associated with an increase in PFS, which is consistent with findings of previous studies investigating the efficacy of PARP inhibitors for platinum sensitive ovarian cancer [32–34]. Based on previous research [35, 36], the efficacy of PARP inhibitors was found to decrease when various chemotherapeutic agents were used, implying that the administration of PARP inhibitors for the early treatment of ovarian cancer could be more beneficial.

PARP inhibitors have a positive effect on both the BRCA mutation positive population and non-BRCA mutation population. However, compared with the non-BRCA, PARP inhibitors have a greater effect than placebo in the BRCA mutation population. In addition, HRD positive status may be the main factor associated with the effects of PARP inhibitors in the non-BRCA mutation population. We found that PARP inhibitors exhibit a clinically significant effect in the HRD positive (including BRCA mutation) population, and HRD positive (excluding BRCA mutation) population. In contrast, no clear differences were found between the groups in the HRD negative population. Although niraparib also showed positive median PFS results in HRD negative population in PRIMA trial [22], there are still concerns that whether it can bring long-term benefit or what is the proper treatment when these patients get relapsed. Therefore, the diagnostic test used to determine HRD status clearly plays an important role in identifying patients who are likely to have improved prognoses following PARP inhibitors maintenance therapy. Although the assessment tools used in the included studies were different, an HRD score of 42 is the commonly adopted cut-off value [37–39]. In the VELIA trial [21], the cut-off value of HRD was 33, which included more participants in the HRD positive population. Nevertheless, the findings of the study were still positive. Further studies are warranted to confirm the optimal cut-off value of HRD.

According to the subgroup analyses on PFS, the effect of PARP inhibitors on ovarian cancer was not affected by FIGO stage status, response to first-line chemotherapy, or residual macroscopic disease after debulking surgery. We observed that PARP inhibitors may have a clinically significant effect in terms of FIGO stage III, with an average decrease in the risk of disease progression or death of 47%. The results of all included studies revealed that PARP inhibitors may lead to an average decrease in the risk of disease progression or death of 36% of FIGO stage IV patients (HR, 0.64; 95% CI 0.48–0.84). However, results of the sensitivity analysis on PARP inhibitors monotherapy (PRIMA [22] and SOLO1 [23]) did not identify any clear difference between PARP inhibitors and placebo (HR, 0.68; 95% CI 0.44–1.05), although the point estimate value was similar to that of the primary analysis. On one hand, the statistically negative findings of the sensitivity analysis may be imprecise due to the insufficient sample size. On the other hand, the results of these two trials were different. The SOLO1 trial [23] included 66 patients with FIGO stage IV, and all of them had a BRCA mutation, whereas, the PRIMA trial [22] included 257 patients with FIGO stage IV, which may have included patients with the non-BRCA mutation as well (specific number not reported). Due to the of significantly larger sample size than that of the SOLO1 trial [23], the PRIMA trial [22] contributed more to the results of the sensitivity analysis, and BRCA mutation seemed to be a confounding factor on this finding, which needs to be elucidated by further research. Clinically significant effect were found in participants with CR to first-line chemotherapy (an average of 54% decrease in risk), those with PR to first-line chemotherapy (an average of 52% decrease in risk), and those with (an average of 41% decrease in risk) and without (an average of 48% decrease in risk) residual macroscopic disease after debulking surgery (also worth noting that, all patients included in this analysis reached complete or partial response after therapy). According to clinical experience, FIGO stage III, without residual macroscopic disease after debulking surgery, and CR to first-line chemotherapy were believed to be the indicators of good prognosis in ovarian cancer.

The results of the current review indicate that PARP inhibitors, as first-line maintenance therapy, result in an increase in the PFS of patients with newly diagnosed ovarian cancer, especially in the HRD positive cohort. A similar trend of benefit was found in some clinical subgroups, even for those patients at high risk who received the rescue therapy, which needs more good-quality evidence before coming to conclusions.

For OS, the results of the present study indicated no clear differences between the groups. This outcome might be due to the lack of evidence, because we only had data from two midterm analyses. Larger trials with longer follow-up are warranted in the future to investigate the effect of PARP inhibitors on the long-term survival of patients with ovarian cancer.

The pooled results found that the effects of PARP inhibitors on HRQoL were similar to those of the placebo. The safety profiles of PARP inhibitors were generally consistent with the previously known AEs associated with each type of drug. Anemia mainly occurred in the olaparib group, and thrombocytopenia occurred in the niraparib group. Hypertension mainly occurred in patients who received bevacizumab, whereas olaparib in combination with bevacizumab did not increase the rates of hypertension. The findings of this systematic review echo the results of each of the relevant RCTs (PAOLA-1 [24], PRIMA [22], SOLO1 [23], and VELIA [21]). Overall AEs did not affect the tolerability of PARP inhibitors and subsequent treatment.

PARP inhibitors are emerging as a promising maintenance therapy that prolongs the PFS of patients with newly diagnosed ovarian cancer. In this review, all the effect estimates were based on single agent in a maintenance context. Further, data on combination therapy and head-to-head comparisons of PARP inhibitors were insufficient. Future trials evaluating factors such as dosage, tolerability, timing, and efficacy are warranted to determine the full potential of PARP inhibitor maintenance therapy in patients with newly diagnosed ovarian cancer [40].

The comparison between PARP inhibitors and placebo was recently investigated in other reviews [41, 42]. PAOLA-1 [24] was not included in the study by Wang 2020, and Gong 2020 mainly focused on different regimens in BRCA-mutated ovarian cancer (newly diagnosed or relapsed) in his study. The present systematic review mainly focused on investigating the survival effect in subgroup populations with specific clinical characteristics. The observed benefits were generally consistent regardless of the FIGO stage, response to chemotherapy, or residual macroscopic disease after debulking surgery. The current review provided more evidence to support use of PARP inhibitors as first-line maintenance therapy in different patients with ovarian cancer.

This systematic review and meta-analysis has several strengths. First, all included RCTs were multicenter studies that included participants from a variety of countries and ethnicities, therefore, the results have high external validity, and might be generalizable to most patients with newly diagnosed ovarian cancer. Second, the quality of evidence is high, and the risk of bias of the included studies is low. Third, the results related to the key outcomes were consistent across RCTs and are unlikely to reduce the quality of evidence despite some statistical heterogeneity. Finally, we used comprehensive search strategies with no limitations on language, date, document type, or publication status. Therefore, it is likely that all relevant trials have been identified. Two reviewers independently conducted screening, data extraction and analysis, thereby minimizing the risk of selection bias and performance bias in the review process.

Nevertheless, this review also has some limitations. First, although the evidence covers several aspects including participants, interventions, and outcomes, and the marked differences among the trials (such as inclusion criteria, mutational status, rate of residual disease after debulking surgery, duration of maintenance therapy), the evidence should be carefully considered when the findings are applied in clinical practice. Second, it was impossible to adjust for all potential confounding factors of interest as our analysis options (such as meta-regression) were limited to the number and sample sizes of the included studies. Due to the fact that there were no data available on rucaparib, the results of this review could not be extended to represent the entire class of PARP inhibitors. Third, heterogeneity was identified in several outcomes, which could be due to differences in the number of patients with a BRCA mutation, HRD status, or type of PARP inhibitor. Lastly, owing to the insufficient data, we were unable to estimate the efficacy of different types of PARP inhibitors or those in combination with bevacizumab and did not perform subgroup analysis of other factors of interest, such as neoadjuvant chemotherapy, used before enrollment. We suggest that all data should be well-reported and expanded in further research, particularly in the subgroups of clinical interest (not only shown in figures or transformed for skewed data).

## Conclusions

In summary, the findings of this systematic review and meta-analysis demonstrate the efficacy of PARP inhibitors in patients with newly diagnosed ovarian cancer. Further, they highlight that the effect of PARP inhibitors on ovarian cancer is probably not affected by FIGO stage status, response to first-line chemotherapy, and residual macroscopic disease after debulking surgery. Compared with placebo, the use of PARP inhibitors as first-line maintenance therapy is associated with the substantial increase benefit of prolonging PFS without negatively impacting on HRQoL. However, we must bear in mind that the diagnostic test used to determine HRD status plays an important role in guiding PARP inhibitor maintenance therapy.

## Supplementary Information

Below is the link to the electronic supplementary material.

Supplementary file1 Supplemental Methods. Search strategies. Supplemental Text. Detailed description on scales which assessing HRQoL. Supplemental Table 1. Data extraction form. Supplemental Table 2. Included RCTs (multi-center) comparing PARPi to placebo in patients with newly diagnosed ovarian cancer. Supplemental Figure 1. 'Risk of bias graph’ (left) and ‘Risk of bias summary’ (right). Supplemental Table 3. Summary of Findings table. Supplemental Figure 2. PARPi versus placebo: Meta-analysis of PFS. Supplemental Figure 3. Sensitivity analysis: PARPi (monotherapy) versus placebo: Meta-analysis of PFS (BRCA and HRD cohort). Supplemental Figure 4. Sensitivity analysis: PARPi (monotherapy) versus placebo: Meta-analysis of PFS (Key subgroups). Supplemental Figure 5. PARPi versus placebo: Meta-analysis of OS. Supplemental Figure 6. PARPi versus placebo: Meta-analysis of HRQoL. Supplemental Table 4. PARPi versus placebo: adverse events (any Grade). Supplemental Table 5. PARPi versus placebo: adverse events (Grade>=3) (DOCX 309 kb)

## Data Availability

This is a systematic review and meta-analysis on available studies by searching electronic database.
