# Supplementary material for: Poly (adenosine diphosphate [ADP]–ribose) polymerase (PARP) inhibitors as maintenance therapy in women with newly diagnosed ovarian cancer: a systematic review and meta-analysis
Source: Arch Gynecol Obstet. 2021 May 21;304(2):285–96. doi: 10.1007/s00404-021-06070-2 (PMC8277645; doi:10.1007/s00404-021-06070-2)
Supplement: Supplementary file 1 — Supplementary file1 Supplemental Methods. Search strategies. Supplemental Text. Detailed description on scales which assessing HRQoL. Supplemental Table 1. Data extraction form. Supplemental Table 2. Included RCTs (multi-center) comparing PARPi to placebo in patients with newly diagnosed ovarian cancer. Supplemental Figure 1. 'Risk of bias graph’ (left) and ‘Risk of bias summary’ (right). Supplemental Table 3. Summary of Findings table. Supplemental Figure 2. PARPi versus placebo: Meta-analysis of PFS. Supplemental Figure 3. Sensitivity analysis: PARPi (monotherapy) versus placebo: Meta-analysis of PFS (BRCA and HRD cohort). Supplemental Figure 4. Sensitivity analysis: PARPi (monotherapy) versus placebo: Meta-analysis of PFS (Key subgroups). Supplemental Figure 5. PARPi versus placebo: Meta-analysis of OS. Supplemental Figure 6. PARPi versus placebo: Meta-analysis of HRQoL. Supplemental Table 4. PARPi versus placebo: adverse events (any Grade). Supplemental Table 5. PARPi versus placebo: adverse events (Grade>=3) (DOCX 309 kb) [file 404_2021_6070_MOESM1_ESM.docx]

# Article title: Poly (adenosine diphosphate [ADP]–ribose) polymerase (PARP)inhibitors as Maintenance Therapy in Women with Newly Diagnosed Ovarian Cancer: A Systematic Review and Meta-analysis

**Journal name:** Archives of Gynecology and Obstetrics

# Author names: Hongyan Cheng; MD; Junjun Yang, PhD; Huixin Liu, PhD; Yang Xiang, MD

# Corresponding author:

# Yang Xiang, Department of Obstetrics and Gynecology, Peking Union Medical College Hospital, Chinese Academy of Medical Sciences and Peking Union Medical College, No.1 Shuaifuyuan, Wangfujing, Dongcheng District, Beijing, 100730, P.R. China;

# Tel.: +86 106 529 6068;

# E-mail: [xiangy@pumch.cn](mailto:xiangy@pumch.cn)

**Supplemental** **Methods. Search strategies**

**Supplemental** **Text. Detailed description on scales which assessing HRQoL**

**Supplemental Table 1. Data extraction form**

**Supplemental Table 2. Included RCTs (multi-center) comparing PARPi to placebo in patients with newly diagnosed ovarian cancer**

**Supplemental Figure 1. 'Risk of bias graph’ (left) and ‘Risk of bias summary’ (right).**

**Supplemental Table 3. Summary of Findings table**

**Supplemental Figure 2. PARPi versus placebo: Meta-analysis of PFS.**

**Supplemental Figure 3. Sensitivity analysis: PARPi (monotherapy) versus placebo: Meta-analysis of PFS (BRCA and HRD cohort).**

**Supplemental Figure 4. Sensitivity analysis: PARPi (monotherapy) versus placebo: Meta-analysis of PFS (Key subgroups).**

**Supplemental Figure 5. PARPi versus placebo: Meta-analysis of OS.**

**Supplemental Figure 6. PARPi versus placebo: Meta-analysis of HRQoL.**

**Supplemental Table 4. PARPi versus placebo: adverse events (any Grade)**

**Supplemental Table 5. PARPi versus placebo: adverse events (Grade>=3)**

**Supplemental Methods. Search strategies**

*A. Cochrane Library*

#1 MeSH descriptor: (Ovarian Neoplasms] explode all trees 1787

#2 ((ovar* NEAR/1 (cancer* or tumor* or tumour* or neoplas* or carcinoma* or adenocarcinoma* or malignan* or oncolog* or sarcoma*))):ti,ab,kw (Word variations have been searched) 6226

#3 #1 or #2 6257

#4 (olaparib or lynparza or AZD2281 or "azd 2281" or "ku 0059436" or "ku 59436" or KU0059436 or KU59436):ti,ab,kw (Word variations have been searched) 350

#5 (niraparib or zejula or "mk 4827" or mk4827):ti,ab,kw (Word variations have been searched) 86

#6 (veliparib or "abt 888" or abt888):ti,ab,kw (Word variations have been searched) 168

#7 #4 or #5 or #6 580

#8 MeSH descriptor: (Placebos] explode all trees 23635

#9 MeSH descriptor: (Placebo Effect] explode all trees 1460

#10 (placebo*):ti,ab,kw (Word variations have been searched) 286986

#11 #8 or #9 or #10 286995

#12 #3 and #7 and #11 106

*B. EMBASE*

1 exp ovary cancer/ (112644)

2 (ovar* adj (cancer* or tumor* or tumour* or neoplas* or carcinoma* or adenocarcinoma* or malignan* or oncolog* or sarcoma*)).tw,kw. (103380)

3 or/1-2 (139431)

4 exp olaparib/ (4171)

5 (olaparib or lynparza or AZD2281 or "azd 2281" or "ku 0059436" or "ku 59436" or KU0059436 or KU59436).tw,kw. (2878)

6 exp niraparib/ (805)

7 (niraparib or zejula or "mk 4827" or mk4827).tw,kw. (594)

8 exp veliparib/ (1626)

9 (veliparib or "abt 888" or abt888).tw,kw. (1358)

10 or/4-9 (5659)

11 3 and 10 (2050)

12 placebo*.tw,kw. (299002)

13 exp placebo/ or exp placebo effect/ (346326)

14 or/12-13 (443316)

15 11 and 14 (334)

16 (clin* adj2 trial).mp. (1517337)

17 ((singl* or doubl* or trebl* or tripl*) adj (blind* or mask*)).mp. (296815)

18 (random* adj5 (assign* or allocat*)).mp. (184137)

19 randomi*.mp. (1171664)

20 crossover.mp. (95413)

21 exp randomized-controlled-trial/ (574747)

22 exp double-blind-procedure/ (166781)

23 exp crossover-procedure/ (61072)

24 exp single-blind-procedure/ (36840)

25 exp randomization/ (84799)

26 or/16-25 (2217429)

27 exp Animals/ or exp Invertebrate/ or Animal Experiment/ or Animal Model/ or Animal Tissue/ or Animal Cell/ or Nonhuman/ (26556827)

28 Human/ or Normal Human/ or Human Cell/ (20293398)

29 27 and 28 (20233668)

30 27 not 29 (6323159)

31 15 and 26 (306)

32 31 not 30 (301)

*C. MEDLINE*

1 exp Ovarian Neoplasms/ (81172)

2 (ovar* adj (cancer* or tumor* or tumour* or neoplas* or carcinoma* or adenocarcinoma* or malignan* or oncolog* or sarcoma*)).tw,kw,kf. (75381)

3 or/1-2 (102474)

4 (olaparib or lynparza or AZD2281 or "azd 2281" or "ku 0059436" or "ku 59436" or KU0059436 or KU59436).tw,kw,kf. (1035)

5 (niraparib or zejula or "mk 4827" or mk4827).tw,kw,kf. (145)

6 (veliparib or "abt 888" or abt888).tw,kw,kf. (318)

7 or/4-6 (1332)

8 3 and 7 (437)

9 placebo*.tw,kw,kf. (208420)

10 exp Placebos/ or exp Placebo Effect/ (38312)

11 or/9-10 (221805)

12 8 and 11 (37)

13 exp Patient Readmission/ or exp Recurrence/ (193077)

14 (discontinu* or halt* or stop* or drop-out* or drop out* or dropout* or rehospitalis* or relaps* or maintain* or maintenance* or recur*).tw,kw,kf. (1733501)

15 or/13-14 (1807610)

16 12 and 15 (36)

17 exp clinical trial/ (837383)

18 exp randomized controlled trials/ (129340)

19 exp double-blind method/ (153515)

20 exp single-blind method/ (27371)

21 exp cross-over studies/ (46073)

22 randomized controlled trial.pt. (490335)

23 clinical trial.pt. (518157)

24 controlled clinical trial.pt. (93274)

25 (clinic* adj2 trial).mp. (700568)

26 (random* adj5 control* adj5 trial*).mp. (696619)

27 (crossover or cross-over).mp. (92631)

28 ((singl* or doubl* or trebl* or tripl*) adj (blind* or mask*)).mp. (228771)

29 randomi*.mp. (847753)

30 (random adj5 (assign* or allocat* or assort*)).mp. (107035)

31 or/17-30 (1350151)

32 16 and 31 (30)

33 animals/ not humans/ (4587142)

34 32 not 33 (30)

*D. Web of Science*

#1 TOPIC: (ovar* NEAR/1 (cancer* or tumor* or tumour* or neoplas* or carcinoma* or adenocarcinoma* or malignan* or oncolog* or sarcoma*)) (110110)

#2 TOPIC: (olaparib or lynparza or AZD2281 or "azd 2281" or "ku 0059436" or "ku 59436" or KU0059436 or KU59436) (1758)

#3 TOPIC: (niraparib or zejula or "mk 4827" or mk4827)

#4 TOPIC: (veliparib or "abt 888" or abt888)

#5 #4 OR #3 OR #2 (2374)

#6 TOPIC: (placebo*) (243291)

#7 #6 AND #5 AND #1 (57)

#8 TOPIC: ("randomized controlled trial" or "controlled clinical trial" or randomized or placebo or "drug therapy" or randomly or trial or groups) (6093757)

#9 #8 AND #7 (57)

*E. The metaRegister of Controlled Trials*

0 Studies found for: text search: Olaparib placebo Ovarian cancer

0 Studies found for: text search: Niraparib placebo Ovarian cancer

0 Studies found for: text search: Veliparib placebo Ovarian cancer

0 Studies found for: text search: Olaparib placebo ovary cancer

0 Studies found for: text search: Niraparib placebo ovary cancer

0 Studies found for: text search: Veliparib placebo ovary cancer

0 Studies found for: text search: Olaparib placebo Ovarian Neoplasm

0 Studies found for: text search: Niraparib placebo Ovarian Neoplasm

0 Studies found for: text search: Veliparib placebo Ovarian Neoplasm

*F. ClinicalTrials.gov*

8 Studies found for: Olaparib and placebo | ovary cancer

6 Studies found for: Niraparib and placebo | ovary cancer

2 Studies found for: Veliparib and placebo | ovary cancer

*G. International Clinical Trials Registry Platform*

15 Studies found for: Olaparib and placebo and ovary cancer

9 Studies found for: Niraparib and placebo and ovary cancer

2 Studies found for: Veliparib and placebo and ovary cancer

**Supplemental Text. Detailed description on scales which assessing HRQoL**

The EORTC QLQ-C30 (using in the PAOLA study) is a cancer-specific questionnaire assessing 15 health-related quality of life (HRQoL) scales through 30-items: a global health status, five functional scales (physical, role, emotional, cognitive, and social) and nine symptomatic scales (fatigue, nausea and vomiting, pain, dyspnea, insomnia, appetite loss, constipation, diarrhea, and financial difficulties). For each dimension, one score is generated on a 0–100 scale, with higher score representing better HRQoL. The between-group difference in the global health status /quality of life dimension of the EORTC QLQ-C30 questionnaire were reported, with a minimal clinically important difference defined as ±10 points.

The FOSI (using in the PRIMA study) is a validated eight-item measure of symptom response to treatment for ovarian cancer based on a subset of questions from the Functional Assessment of Cancer Therapy - Ovarian Cancer questionnaire. For each question, patients responded to their symptom experience over the previous 7 days using a five-point Likert scale of “not at all” (0) to “very much” (4). The FOSI score range is 0 (severely symptomatic) to 32 (asymptomatic). The FOSI score was derived in accordance with the FOSI scoring manual. Negatively stated items are reversed by subtracting the response from “4”. After reversing proper items, all subscale items are summed to a total, which is the FOSI score. A higher score indicates a better quality of life (QOL). If there are missing items, subscale scores will be prorated as long as more than 50% of the items were answered (ie, at least 5 of 8 items). The proration is done by multiplying the sum of the subscale by the number of items in the subscale, then dividing by the number of items actually answered. FOSI score = [Sum of item scores] x 8 / [N of items answered]

The Functional Assessment of Cancer Therapy – Ovarian Cancer (FACT-O) Questionnaire (using in the SOLO1 study) is a reliable and well validated instrument to assess health-related quality of life (HRQoL) in women with ovarian cancer. The Trial Outcome Index (TOI) is a summary index of physical and functional well-being and key ovarian cancer symptoms derived from the FACT-O questionnaire (TOI scores range from 0 to 100, with higher scores indicating better HRQoL).

The Disease Related Symptom score is a subset of the National Comprehensive Cancer Network Functional Assessment of Cancer Therapy Ovarian Symptom Index–18 (NFOSI-18) (using in the VELIA study), which evaluates nine symptoms related to disease or treatment. This questionnaire was administered at protocol-defined intervals until disease progression or up to 2 years after the receipt of the first dose, whichever was later. Scores range from 0 to 36, with higher scores indicating a lower burden of symptoms. A 3-point difference was defined as clinically meaningful.

**Supplemental Table 1. Data extraction form**

| **Study ID** | |
| --- | --- |
| **Contact Info** | |
| **Methods** | Country/Location |
|  | Setting (Inpatient/Outpatient/Community/NR] |
|  | Random (Parallel/Cross-over/Cluster] |
|  | Blind (Single/Double/Open-label/NR] |
|  | Centre (Single/Multi/NR] |
| **Participants** | Diagnosis |
|  | Diagnositic criteria |
|  | Stage of disease |
|  | Sample size at randomisation |
|  | Mean age (years) |
|  | Min age (years) |
|  | Max age (year) |
|  | ECOG score |
|  | BRCA‑mutation status (Yes/No/Unknown] |
|  | Homologous‑recombination deficiency (Positive/Negative/Unknown] |
|  | FIGO stage (III/IV] |
|  | Presence of residual macroscopic disease after debulking surgery performed before trial entry (Yes/No] |
|  | Chemotherapy before enrollment |
|  | Response to first-line chemotherapy (CR/PR] |
|  | Inclusion criteria |
|  | Exclusion criteria |
| **Interventions** | Sample size of PARPi group at randomisation |
|  | Description of PARPi |
|  | Dosage and frequency of PARPi |
|  | Duration of PARPi |
|  | Sample size of Placebo group at randomisation |
|  | Description of Placebo |
|  | Description of other drugs combination (Same in two compared groups] |
|  | Monoclonal antibody agent combination (Yes/No] |
| **Outcomes** | Outcome name |
|  | Defination/Meaurement of outcome |
|  | Timepoint of assessment |
|  | Outcomes (unable to use/not predefined in protocol) |
| **Outcome data** | |
| **Time to event data** (such as PFS/OS) | HR |
|  | Lower limit of 95% CI |
|  | Higher limit of 95% CI |
| **Dichotomous data** (such as AE) | Events number in PARPi group |
|  | Total number in PARPi group |
|  | Events number in placebo group |
|  | Total number in placebo group |
| **Continuous data** (such as QoL) | Mean of PARPi group |
|  | SD of PARPi group |
|  | Total number in PARPi group |
|  | Mean of placebo group |
|  | SD of placebo group |
|  | Total number in placebo group |
| **ROB assessment of included studies** | |
| **Randomisation** | Support for judgement |
| **Allocation concealment** | Support for judgement |
| **Blinding of participants and personnel** | Support for judgement |
| **Blinding of outcome assessor** | Support for judgement |
| **Incomplete data** | Support for judgement (drop-out) |
| **Selective reporting** | Support for judgement |
| **Other bias** | Support for judgement (study funding) |

**Supplemental Table 2. Included RCTs (multi-center) comparing PARPi to placebo in patients with newly diagnosed ovarian cancer**

| **Author with publication year** (Trial ID) | **CA-125 status (**(No. (%)) **(criteria of assessment** ^a^**, status)** | **Criteria for HRD** | **Chemotherapy before enrollment** |
| --- | --- | --- | --- |
| Ray-Coquard 2019 ^b^ (PAOLA-1, NCT02477644) | Normal level 697 (86.5) Abnormal level 108 (13.4) Missing data 1 (0.1)  CR1/PR1 ^a^ | Tumor HRD status was determined with the use of the myChoice HRD Plus assay ^c^ | 6 to 9 cycles of platinum-taxane based chemotherapy |
| Moore 2018 (SOLO1, NCT01844986) | ≤ULN 370 (94.6) >ULN 20 (5.1) Missing data 1 (0.3)  CR2/PR2 ^a^ | Not reported | 4 to 9 cycles of platinum-based chemotherapy without bevacizumab |
| González‑Martín 2019 (PRIMA, NCT02655016) | Normal or >90% CA-125 following front-line platinum treatment (no details reported) | Tumor HRD status was determined with the use of the myChoice HRD Plus assay ^d^ | 6 to 9 cycles of first-line platinum-based chemotherapy |
| Coleman 2019^d^ (VELIA, NCT02470585) | CR3/Progression (no details reported) | the myChoice assay ^e^ | platinum-based chemotherapy: carboplatin/paclitaxel |
| Abbreviations: CA-125: cancer antigen 125; CR: complete response; PR: partial response; ULN: upper limit of the normal range.  a the criteria of assessment: 1) CR1: the disappearance of all measurable or assessable disease and normalization of CA-125 levels; PR1: radiologic evidence of disease, an abnormal CA-125 level, or both; 2) CR2: no evidence of disease on imaging after chemotherapy and a normal CA-125 level; PR2: >=30% decrease in tumor volume from the start to the end of chemotherapy or no evidence of disease on imaging after chemotherapy but a CA-125 level above the upper limit of the normal range; 3) CR3: if CA-125 is initially above the upper normal limit, it must normalize for a subject to be considered in CR; Progression: in the absence of radiographic or clinical evidence of progressive disease, a rise in CA-125 alone is not sufficient to declare progression.  b Bevacizumab combined use in both olaparib and placebo groups.  c myChoice HRD Plus assay (Myriad Genetic Laboratories): an HRD score of 42 or higher indicated a positive test, and an HRD score of less than 42 indicated a negative test.  d Three compared groups in this RCT: 1) control group: chemotherapy plus placebo followed by placebo maintenance; 2) veliparib-combination-only group: chemotherapy plus veliparib followed by placebo maintenance; 3) veliparib-throughout group: chemotherapy plus veliparib followed by veliparib maintenance.  e myChoice assay: on which a score of ≥33 was considered to indicate HRD status, and a score of <33 was considered to indicate non-HRD status; the threshold score was revised from 42, after several retrospective analyses of previous clinical trials, to increase the sensitivity of detecting a response to PARP inhibitors. | | | |

**Supplemental Figure 1. 'Risk of bias graph’ (left) and ‘Risk of bias summary’ (right).**


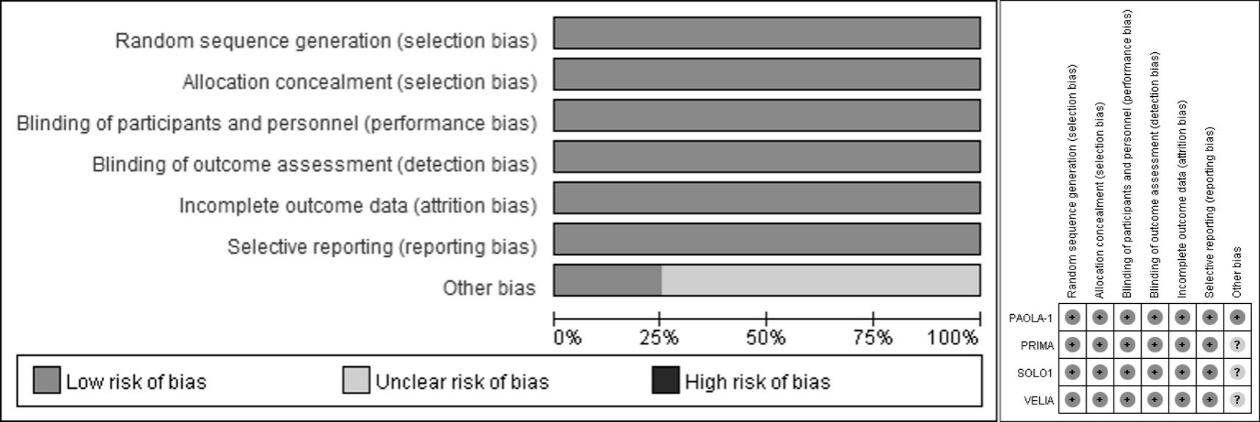


**Supplemental Table 3. Summary of Findings table**

| **PARPi compared to Placebo for Newly Diagnosed Ovarian Cancer** | | | | | | | |
| --- | --- | --- | --- | --- | --- | --- | --- |
| **Patient or population**: Newly Diagnosed Ovarian Cancer  **Setting**: clinical complete response or partial response following completion of first line platinum-based chemotherapy  **Intervention**: PARPi  **Comparison**: Placebo | | | | | | | |
| Outcomes | **Anticipated absolute effects^*^** (95% CI) | | Relative effect (95% CI) | № of participants  (studies) | Certainty of the evidence (GRADE) | Comments | |
|  | **Risk with Placebo** | **Risk with PARPi** |  |  |  |  |  |
| Progress-free survival (PFS) assessed by investigator [progression or death] Follow-up: range 2 to 3 years | **with BRCA mutation** | | **HR 0.34** (0.28 to 0.41) | 1051 (4 RCTs) | ⨁⨁⨁◯ Moderate ^a^ | PARPi probably results in large increase in PFS. | |
|  | 631 per 1,000 | **289 per 1,000** (214 to 258) |  |  |  |  |  |
|  | **without BRCA mutation** | | **HR 0.75** (0.64 to 0.87) | 1068 (2 RCTs) | ⨁⨁⨁⨁ High ^a^ | PARPi results in moderate increase in PFS. | |
|  | 713 per 1,000 | **534 per 1,000** (456 to 620) |  |  |  |  |  |
|  | **HRD positive (including BRCA mutation)** | | **HR 0.39** (0.29 to 0.53) | 1572 (4 RCTs) | ⨁⨁⨁◯ Moderate ^a^ | PARPi probably results in large increase in PFS. | |
|  | 645 per 1,000 | **251 per 1,000** (187 to 342) |  |  |  |  |  |
|  | **HRD positive (excluding BRCA mutation)** | | **HR 0.46** (0.33 to 0.63) | 302 (2 RCTs) | ⨁⨁⨁◯ Moderate ^a^ | PARPi probably results in large or moderate increase in PFS. | |
|  | 664 per 1,000 | **394 per 1,000** (302 to 497) |  |  |  |  |  |
|  | **HRD negative** | | **HR 0.83** (0.67 to 1.03) | 775 (3 RCTs) | ⨁⨁◯◯ Low ^a,b^ | PARPi may have little or no effect in PFS. | |
|  | 730 per 1,000 | **605 per 1,000** (489 to 752) |  |  |  |  |  |
|  | **FIGO III** | | **HR 0.53** (0.39 to 0.71) | 1952 (4 RCTs) | ⨁⨁⨁◯ Moderate ^a^ | PARPi probably results in large or moderate increase in PFS. | |
|  | 661 per 1,000 | **350 per 1,000** (257 to 469) |  |  |  |  |  |
|  | **FIGO IV** | | **HR 0.64** (0.48 to 0.84) | 734 (4 RCTs) | ⨁⨁⨁◯ Moderate ^a^ | PARPi probably results in moderate increase in PFS. | |
|  | 688 per 1,000 | **440 per 1,000** (330 to 578) |  |  |  |  |  |
|  | **CR to first-line chemotherapy** | | **HR 0.46** (0.32 to 0.65) | 1006 (3 RCTs) | ⨁⨁⨁⨁ High | PARPi probably results in large increase in PFS. | |
|  | 648 per 1,000 | **381 per 1,000** (284 to 493) |  |  |  |  |  |
|  | **PR to first-line chemotherapy** | | **HR 0.48** (0.23 to 0.99) | 511 (3 RCTs) | ⨁⨁⨁◯ Moderate ^b^ | PARPi probably results in large or slight increase in PFS. | |
|  | 798 per 1,000 | **536 per 1,000** (308 to 795) |  |  |  |  |  |
|  | **with presence of residual macroscopic disease after debulking surgery performed before trial entry** | | **HR 0.59** (0.47 to 0.73) | 507 (3 RCTs) | ⨁⨁⨁◯ Moderate ^a^ | PARPi probably results in moderate increase in PFS. | |
|  | 746 per 1,000 | **440 per 1,000** (350 to 544) |  |  |  |  |  |
|  | **without presence of residual macroscopic disease after debulking surgery performed before trial entry** | | **HR 0.52** (0.34 to 0.81) | 1133 (3 RCTs) | ⨁⨁⨁◯ Moderate ^a^ | PARPi probably results in large or moderate increase in PFS. | |
|  | 629 per 1,000 | **327 per 1,000** (214 to 510) |  |  |  |  |  |
| Overall survival (OS) [death] Follow-up: range 2 to 3 years | **Overall population** | | **HR 0.82** (0.59 to 1.13) | 1124 (2 RCTs) | ⨁⨁◯◯ Low ^b,c^ | PARPi may have little or no effect in OS but important effects cannot be confirmed or excluded due to data immaturity and crossover of the following treatment. | |
|  | 200 per 1,000 | **167 per 1,000** (123 to 223) |  |  |  |  |  |
| Health-related Quality of life (HRQoL)  assessed with: change score from the baseline, score of scales, high = well, scale from: 0 to 100)  Follow up: 2 years | - | SMD **0.12 lower** (0.6 lower to 0.36 higher) | - | 1106 (2 RCTs) | ⨁⨁⨁⨁ High | PARPi does not have clinically important impact on HRQoL. | |
| ***The risk in the intervention group** (and its 95% confidence interval) is based on the assumed risk in the comparison group and the **relative effect** of the intervention (and its 95% CI).   **CI:** Confidence interval; **HR:** Hazard Ratio; **OS:** Overall Survival; **PFS:** Progression Free Survival; **SMD:** Standardized mean difference; | | | | | | | |
| **GRADE Working Group grades of evidence** **High certainty:** We are very confident that the true effect lies close to that of the estimate of the effect **Moderate certainty:** We are moderately confident in the effect estimate: The true effect is likely to be close to the estimate of the effect, but there is a possibility that it is substantially different **Low certainty:** Our confidence in the effect estimate is limited: The true effect may be substantially different from the estimate of the effect **Very low certainty:** We have very little confidence in the effect estimate: The true effect is likely to be substantially different from the estimate of effect | | | | | | | |
| Explanations ^a^ Downgraded by one level due to selection bias: subgroup data from included RCTs were used. (We did not downgrade the evidence when the subgroup factor was considered in the process of randomization.)  ^b^ Downgraded by one level due to imprecision: very wide CIs.  ^c^ Downgraded by one level due to attrition bias on this outcome (overall survival): data are immature in two RCTs | | | | | | |  |

**Supplemental Figure 2. PARPi versus placebo: Meta-analysis of PFS.**


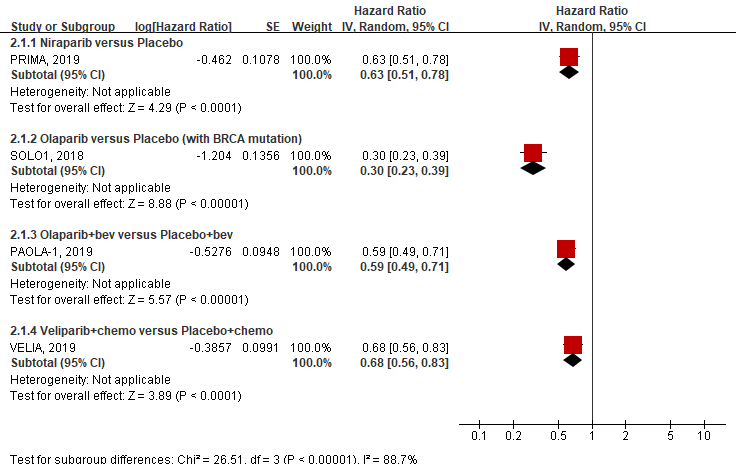


**Supplemental Figure 3. Sensitivity analysis: PARPi (monotherapy) versus placebo: Meta-analysis of PFS (BRCA and HRD cohort).**

**
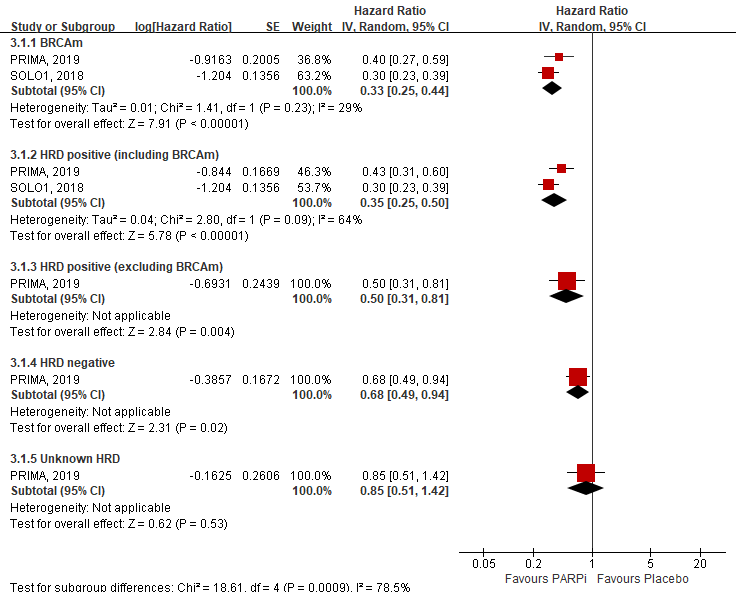
**

**Supplemental Figure 4. Sensitivity analysis: PARPi (monotherapy) versus placebo: Meta-analysis of PFS (Key subgroups).**

**
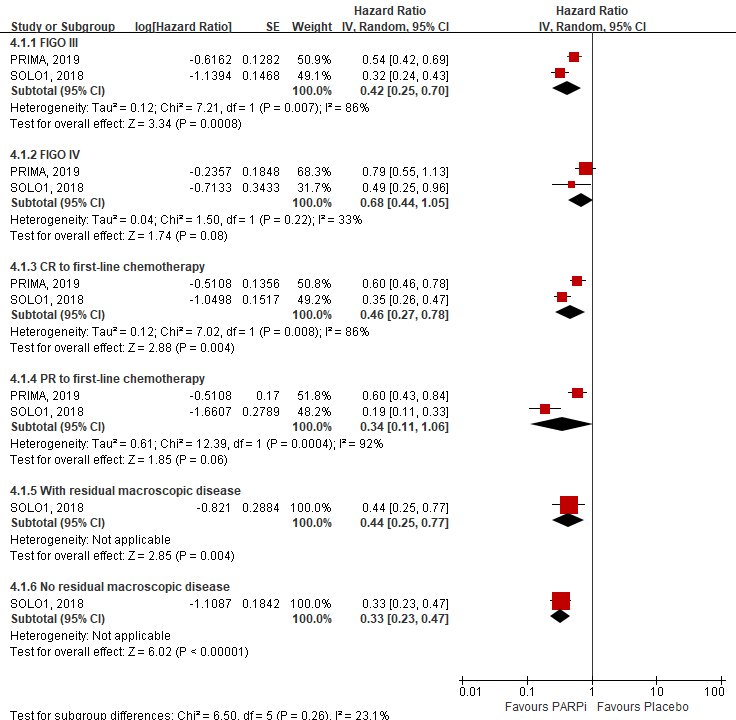
**

**Supplemental Figure 5. PARPi versus placebo:** **Meta-analysis of OS.**


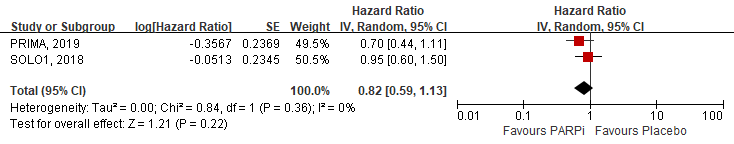


**Supplemental Figure 6. PARPi versus placebo: Meta-analysis of HRQoL**.


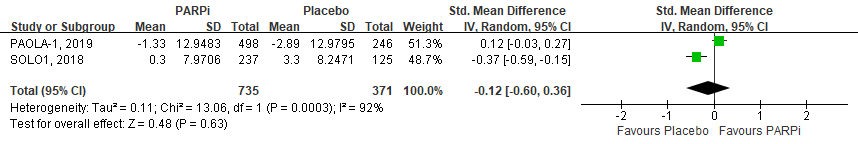


**Supplemental Table 4. PARPi versus placebo: adverse events (all Grade)**

| **Adverse event** | **PAOLA-1 No. of Events (%)** | | **SOLO1 No. of Events (%)** | | **PRIMA No. of Events (%)** | | **VELIA No. of Events (%)** | |
| --- | --- | --- | --- | --- | --- | --- | --- | --- |
|  | **Olaparib+bevacizumab (n=535)** | **Placebo+bevacizumab (n=267)** | **Olaparib (n=260)** | **Placebo (n=130)** | **Niraparib (n=484)** | **Placebo (n=244)** | **Veliparib (n=310)** | **Placebo (n=308)** |
| abdominal distension | NR | NR | NR | NR | 32 (6.61)^a^ | 30 (12.3) | NR | NR |
| abdominal pain | 103 (19.25) | 53 (19.85) | 110 (42.31) | 42 (32.31) | 106 (21.9)^a^ | 75 (30.74) | 56 (18.06) | 45 (14.61) |
| acute lymphocytic leukaemia | 1 (0.19) | 0 (0) | NR | NR | NR | NR | NR | NR |
| acute pyelonephritis | 1 (0.19) | 0 (0) | NR | NR | NR | NR | NR | NR |
| anemia | 219 (40.93) | 27 (10.11)^b^ | 101 (38.85) | 13 (10)^b^ | 307 (63.43) | 43 (17.62)^b^ | 51 (16.45) | 41 (13.31) |
| arthralgia | 116 (21.68) | 64 (23.97) | 66 (25.38) | 35 (26.92) | 85 (17.56) | 47 (19.26) | 50 (16.13) | 51 (16.56) |
| asthenia | NR | NR | NR | NR | 78 (16.12) | 31 (12.7) | NR | NR |
| back pain | 33 (6.17) | 9 (3.37) | 40 (15.38) | 16 (12.31) | 64 (13.22) | 24 (9.84) | 30 (9.68) | 31 (10.06) |
| balance disorder | 0 (0) | 1 (0.37) | NR | NR | NR | NR | NR | NR |
| blood creatinine increased | 28 (5.23) | 3 (1.12)^b^ | NR | NR | 55 (11.36) | 10 (4.1)^b^ | NR | NR |
| breast cancer | 2 (0.37) | 2 (0.75) | 3 (1.15) | 3 (2.31) | NR | NR | NR | NR |
| cardiac failure | 2 (0.37) | 0 (0) | NR | NR | NR | NR | NR | NR |
| constipation | 53 (9.91) | 28 (10.49) | 72 (27.69) | 25 (19.23) | 189 (39.05) | 46 (18.85)^b^ | 36 (11.61) | 42 (13.64) |
| coronary artery disease | 4 (0.75) | 2 (0.75) | NR | NR | NR | NR | NR | NR |
| cough | NR | NR | 42 (16.15) | 28 (21.54) | 74 (15.29) | 35 (14.34) | NR | NR |
| cystitis | 20 (3.74) | 16 (5.99) | NR | NR | NR | NR | NR | NR |
| decreased appetite | 42 (7.85) | 9 (3.37)^b^ | 51 (19.62) | 13 (10)^b^ | 92 (19.01) | 20 (8.2)^b^ | 35 (11.29) | 11 (3.57)^b^ |
| diarrhoea | 98 (18.32) | 45 (16.85) | 89 (34.23) | 32 (24.62) | 91 (18.8) | 55 (22.54) | 58 (18.71) | 41 (13.31) |
| dizziness or vertigo | 28 (5.23) | 12 (4.49) | 51 (19.62) | 20 (15.38) | 71 (14.67) | 26 (10.66) | 38 (12.26) | 23 (7.47) |
| dysgeusia | 42 (7.85) | 3 (1.12)^b^ | 68 (26.15) | 5 (3.85)^b^ | NR | NR | NR | NR |
| dyspepsia | NR | NR | 43 (16.54) | 16 (12.31) | NR | NR | NR | NR |
| dyspnea | 42 (7.85) | 9 (3.37)^b^ | 39 (15) | 7 (5.38)^b^ | 88 (18.18) | 30 (12.3)^b^ | NR | NR |
| edema | 40 (7.48) | 18 (6.74) | NR | NR | NR | NR | NR | NR |
| erythema | 16 (2.99) | 1 (0.37)^b^ | NR | NR | NR | NR | NR | NR |
| erythematous rash | 1 (0.19) | 0 (0) | NR | NR | NR | NR | NR | NR |
| fatigue/asthenia | 283 (52.9) | 86 (32.21)^b^ | 165 (63.46) | 54 (41.54)^b^ | 246 (50.83) | 103 (42.21)^b^ | 71 (22.9) | 53 (17.21) |
| headache | 73 (13.64) | 36 (13.48) | 59 (22.69) | 31 (23.85) | 126 (26.03) | 36 (14.75)^b^ | 32 (10.32) | 29 (9.42) |
| head and neck cancer | NR | NR | 1 (0.38) | 0 (0) | NR | NR | NR | NR |
| hot flush | NR | NR | NR | NR | 54 (11.16) | 20 (8.2) | NR | NR |
| hypertension | 245 (45.79)^a^ | 160 (59.93) | NR | NR | 82 (16.94) | 17 (6.97)^b^ | NR | NR |
| insomnia | NR | NR | NR | NR | 119 (24.59) | 35 (14.34)^b^ | 40 (12.9) | 27 (8.77) |
| intestinal obstruction | 21 (3.93) | 9 (3.37) | NR | NR | NR | NR | NR | NR |
| kidney infection | 1 (0.19) | 0 (0) | NR | NR | NR | NR | NR | NR |
| leukopenia | 95 (17.76) | 26 (9.74)^b^ | NR | NR | 131 (27.07) | 21 (8.61)^b^ | 32 (10.32) | 13 (4.22)^b^ |
| lymphopenia | 126 (23.55) | 25 (9.36)^b^ | NR | NR | NR | NR | NR | NR |
| lung cancer | 1 (0.19) | 0 (0) | NR | NR | NR | NR | NR | NR |
| MDS/AML/AA | 6 (1.12) | 1 (0.37) | 3 (1.15) | 1 (0.77) | 1 (0.21) | 0 (0) | 1 (0.32) | 0 (0) |
| muscle spasms | 17 (3.18) | 12 (4.49) | NR | NR | NR | NR | NR | NR |
| myeloma | 1 (0.19) | 0 (0) | NR | NR | NR | NR | NR | NR |
| nausea | 285 (53.27) | 58 (21.72)^b^ | 201 (77.31) | 49 (37.69)^b^ | 278 (57.44) | 67 (27.46)^b^ | 172 (55.48) | 59 (19.16)^b^ |
| neck pain | 10 (1.87) | 8 (3) | NR | NR | NR | NR | NR | NR |
| neutropenia | 95 (17.76) | 42 (15.73) | 60 (23.08) | 15 (11.54)^b^ | 210 (43.39) | 21 (8.61)^b^ | 51 (16.45) | 38 (12.34) |
| neutrophil count decreased | NR | NR | NR | NR | 82 (16.94) | 5 (2.05)^b^ | NR | NR |
| pancreatic cancer | 1 (0.19) | 0 (0) | NR | NR | NR | NR | NR | NR |
| peripheral sensory neuropathy | 59 (11.03) | 18 (6.74) | NR | NR | NR | NR | 50 (16.13) | 44 (14.29) |
| platelet count decreased | NR | NR | NR | NR | 133 (27.48) | 3 (1.23)^b^ | NR | NR |
| pneumonitis/ILD | 6 (1.12) | 0 (0) | 5 (1.92) | 0 (0) | NR | NR | NR | NR |
| proteinuria | 31 (5.79)^a^ | 40 (14.98) | NR | NR | NR | NR | NR | NR |
| pruritic rash | 1 (0.19) | 0 (0) | NR | NR | NR | NR | NR | NR |
| pyelonephritis | 4 (0.75) | 0 (0) | NR | NR | NR | NR | NR | NR |
| pyuria | 0 (0) | 1 (0.37) | NR | NR | NR | NR | NR | NR |
| rash | 17 (3.18) | 12 (4.49) | NR | NR | NR | NR | NR | NR |
| renal impairment | 32 (5.98) | 4 (1.5)^b^ | NR | NR | NR | NR | NR | NR |
| sleep disorder | 19 (3.55) | 10 (3.75) | NR | NR | NR | NR | NR | NR |
| squamous skin cancer | 1 (0.19) | 0 (0) | NR | NR | NR | NR | NR | NR |
| stomatitis or pharyngitis | 34 (6.36) | 9 (3.37) | NR | NR | NR | NR | NR | NR |
| thrombocytopenia | 42 (7.85) | 9 (3.37)^b^ | 29 (11.15) | 5 (3.85)^b^ | 355 (73.35) | 12 (4.92)^b^ | 61 (19.68) | 23 (7.47)^b^ |
| thyroid cancer | 0 (0) | 1 (0.37) | 1 (0.38) | 0 (0) | NR | NR | NR | NR |
| umbilical erythema | 1 (0.19) | 0 (0) | NR | NR | NR | NR | NR | NR |
| upper abdominal pain | NR | NR | 46 (17.69) | 17 (13.08) | NR | NR | NR | NR |
| upper respiratory tract infection | 31 (5.79) | 14 (5.24) | NR | NR | 49 (10.12) | 25 (10.25) | NR | NR |
| urinary tract infection | 79 (14.77) | 27 (10.11) | NR | NR | NR | NR | NR | NR |
| urosepsis | 0 (0) | 1 (0.37) | NR | NR | NR | NR | NR | NR |
| vomiting | 117 (21.87) | 29 (10.86)^b^ | 104 (40) | 19 (14.62)^b^ | 108 (22.31) | 29 (11.89)^b^ | 105 (33.87) | 30 (9.74)^b^ |
| white blood cell count decreased | NR | NR | NR | NR | 74 (15.29) | 8 (3.28)^b^ | NR | NR |
| musculoskeletal pain | 62 (11.59) | 28 (10.49) | NR | NR | NR | NR | NR | NR |

^a^ P<0.05 (favor PARPi); ^b^ P<0.05 (favor placebo)

**Supplemental Table 5. PARPi versus placebo: adverse events (Grade>=3)**

| **Adverse event** | **PAOLA-1 No. of Events (%)** | | **SOLO1 No. of Events (%)** | | **PRIMA No. of Events (%)** | | **VELIA No. of Events (%)** | |
| --- | --- | --- | --- | --- | --- | --- | --- | --- |
|  | **Olaparib+bevacizumab (n=535)** | **Placebo+bevacizumab (n=267)** | **Olaparib (n=260)** | **Placebo (n=130)** | **Niraparib (n=484)** | **Placebo (n=244)** | **Veliparib (n=310)** | **Placebo (n=308)** |
| abdominal pain | 8 (1.5) | 5 (1.87) | 4 (1.54) | 1 (0.77) | 7 (1.45) | 1 (0.41) | 9 (2.9) | 2 (0.65) |
| anemia | 93 (17.38) | 1 (0.37)^b^ | 56 (21.54) | 2 (1.54)^b^ | 150 (30.99) | 4 (1.64)^b^ | 23 (7.42) | 10 (3.25)^b^ |
| arthralgia | 3 (0.56) | 4 (1.5) | NR | NR | 2 (0.41) | 0 (0) | 2 (0.65) | 0 (0) |
| asthenia | NR | NR | NR | NR | 4 (0.83) | 2 (0.82) | NR | NR |
| back pain | NR | NR | NR | NR | NR | NR | 2 (0.65) | 1 (0.32) |
| blood creatinine increased | NR | NR | NR | NR | 1 (0.21) | 0 (0) | NR | NR |
| cardiac failure | 1 (0.19) | 0 (0) | NR | NR | NR | NR | NR | NR |
| coronary artery disease | 2 (0.37) | 1 (0.37) | NR | NR | NR | NR | NR | NR |
| edema | 1 (0.19) | 0 (0) | NR | NR | NR | NR | NR | NR |
| constipation | 0 (0) | 1 (0.37) | NR | NR | 1 (0.21) | 0 (0) | 0 (0) | 2 (0.65) |
| cough | NR | NR | NR | NR | 0 (0) | 1 (0.41) | NR | NR |
| decreased appetite | 1 (0.19) | 1 (0.37) | NR | NR | 3 (0.62) | 0 (0) | 1 (0.32) | 1 (0.32) |
| diarrhoea | 12 (2.24) | 5 (1.87) | 8 (3.08) | 0 (0) | 3 (0.62) | 1 (0.41) | 1 (0.32) | 1 (0.32) |
| dizziness or vertigo | 2 (0.37) | 1 (0.37) | 0 (0) | 1 (0.77) | 0 (0) | 1 (0.41) | 2 (0.65) | 0 (0) |
| dysgeusia | 1 (0.19) | 0 (0) | NR | NR | NR | NR | NR | NR |
| stomatitis or pharyngitis | 1 (0.19) | 0 (0) | NR | NR | NR | NR | NR | NR |
| intestinal obstruction | 12 (2.24) | 6 (2.25) | NR | NR | NR | NR | NR | NR |
| dyspnea | 5 (0.93) | 1 (0.37) | NR | NR | 2 (0.41) | 2 (0.82) | NR | NR |
| fatigue/asthenia | 28 (5.23) | 4 (1.5)^b^ | 10 (3.85) | 2 (1.54) | 13 (2.69) | 3 (1.23) | 19 (6.13) | 6 (1.95)^b^ |
| headache | 2 (0.37) | 2 (0.75) | 1 (0.38) | 3 (2.31) | 2 (0.41) | 0 (0) | 1 (0.32) | 0 (0) |
| hot flush | NR | NR | NR | NR | 1 (0.21) | 0 (0) | NR | NR |
| hypertension | 100 (18.69)^a^ | 81 (30.34) | NR | NR | 29 (5.99) | 3 (1.23)^b^ | NR | NR |
| insomnia | NR | NR | NR | NR | 4 (0.83) | 1 (0.41) | 3 (0.97) | 1 (0.32) |
| leukopenia | 10 (1.87) | 4 (1.5) | NR | NR | 22 (4.55) | 0 (0)^b^ | 3 (0.97) | 1 (0.32) |
| lymphopenia | 38 (7.1) | 3 (1.12)^b^ | NR | NR | NR | NR | NR | NR |
| sleep disorder | 1 (0.19) | 0 (0) | NR | NR | NR | NR | NR | NR |
| rash | 0 (0) | 1 (0.37) | NR | NR | NR | NR | NR | NR |
| nausea | 13 (2.43) | 2 (0.75) | 2 (0.77) | 0 (0) | 6 (1.24) | 2 (0.82) | 16 (5.16) | 2 (0.65)^b^ |
| neutropenia | 32 (5.98) | 8 (3) | 22 (8.46) | 6 (4.62) | 99 (20.45) | 3 (1.23)^b^ | 16 (5.16) | 6 (1.95)^b^ |
| neutrophil count decreased | NR | NR | NR | NR | 37 (7.64) | 0 (0)^b^ | NR | NR |
| peripheral sensory neuropathy | 3 (0.56) | 3 (1.12) | NR | NR | NR | NR | 2 (0.65) | 1 (0.32) |
| platelet count decreased | NR | NR | NR | NR | 63 (13.02) | 0 (0)^b^ | NR | NR |
| thrombocytopenia | 9 (1.68) | 1 (0.37) | 2 (0.77) | 2 (1.54) | 202 (41.74) | 1 (0.41)^b^ | 20 (6.45) | 4 (1.3)^b^ |
| vomiting | 8 (1.5) | 5 (1.87) | 1 (0.38) | 1 (0.77) | 4 (0.83) | 2 (0.82) | 5 (1.61) | 3 (0.97) |
| white blood cell count decreased | NR | NR | NR | NR | 12 (2.48) | 0 (0) | NR | NR |
| proteinuria | 5 (0.93) | 1 (0.37) | NR | NR | NR | NR | NR | NR |
| musculoskeletal pain | 5 (0.93) | 1 (0.37) | NR | NR | NR | NR | NR | NR |
| urinary tract infection | 1 (0.19) | 1 (0.37) | NR | NR | NR | NR | NR | NR |

^a^ P<0.05 (favor PARPi); ^b^ P<0.05 (favor placebo)
